# Ultra-High Density SNParray in Neuroblastoma Molecular Diagnostics

**DOI:** 10.3389/fonc.2014.00202

**Published:** 2014-08-12

**Authors:** Inge M. Ambros, Clemens Brunner, Reza Abbasi, Christian Frech, Peter F. Ambros

**Affiliations:** ^1^Children’s Cancer Research Institute, St. Anna Kinderkrebsforschung, Vienna, Austria; ^2^Department of Pediatrics, Medical University of Vienna, Vienna, Austria

**Keywords:** SNParray, neuroblastoma, genomic, genetic risk factors, amplification, chromothripsis, precision medicine, INRG

## Abstract

Neuroblastoma serves as a paradigm for applying tumor genomic data for determining patient prognosis and thus for treatment allocation. *MYCN* status, i.e., amplified vs. non-amplified, was one of the very first biomarkers in oncology to discriminate aggressive from less aggressive or even favorable clinical courses of neuroblastoma. However, *MYCN* amplification is by far not the only genetic change associated with unfavorable clinical courses. So called “segmental chromosomal aberrations,” (SCAs) i.e., gains or losses of chromosomal fragments, can also indicate tumor aggressiveness. The clinical use of these genomic aberrations has, however, been hampered for many years by methodical and interpretational problems. Only after reaching worldwide consensus on markers, methodology, and data interpretation, information on SCAs has recently been implemented in clinical studies. Now, a number of collaborative studies within COG, GPOH, and SIOPEN use genomic information to stratify therapy for patients with localized and metastatic disease. Recently, new types of DNA based aberrations influencing the clinical behavior of neuroblastomas have been described. Deletions or mutations of genes like *ATRX* and a phenomenon referred to as “chromothripsis” are all assumed to correlate with an unfavorable clinical behavior. However, these genomic aberrations need to be scrutinized in larger studies applying the most appropriate techniques. Single nucleotide polymorphism arrays have proven successful in deciphering genomic aberrations of cancer cells; these techniques, however, are usually not applied in the daily routine. Here, we present an ultra-high density (UHD) SNParray technique which is, because of its high specificity and sensitivity and the combined copy number and allele information, highly appropriate for the genomic diagnosis of neuroblastoma and other malignancies.

## Introduction

Precision medicine aims to adjust the therapy of cancer patients according to the predicted biological behavior and/or genomic profile of the individual tumor, which can be assessed by different methods. The use of biomarkers is especially important for neuroblastoma patients, as the clinical and biological behavior may vary drastically. A subset of neuroblastic tumors will undergo spontaneous regression (especially in infants) or spontaneous maturation (in children), whereas other neuroblastomas will rapidly progress despite intensive multimodal therapy. This clinical heterogeneity has been common knowledge for decades (1–4), and for many years only age and tumor stage (currently the disease is divided into localized stages: L1 and L2, depending on the resectability, and disseminated stages: M and MS, the latter representing the former 4S stage (5)) were used to categorize neuroblastoma patients. However, in most cases this information alone cannot reliably predict tumor behavior. Over the past three decades, tumor histology (6, 7), status of the *MYCN* oncogene (8, 9), and tumor cell DNA content (ploidy) (10, 11) have each been shown to be independently predictive of patient outcome in large retrospective and prospective studies. Besides these two genomic markers, also a number of segmental chromosomal aberrations (SCAs), affecting different chromosomes, have been described and it is now assumed that, besides *MYCN* amplification, the presence of these aberrations does also have a strong effect on the tumor cells, rendering them more aggressive and/or therapy-refractory (12–14). However, recent data indicate that the overall survival (OS) is not influenced by the presence of SCAs in patients below 18 months with localized resectable, not *MYCN* amplified disease. On the other hand, SCAs are associated with unfavorable OS in >18 months patients with the same clinical feature (Ambros et al., personal observation). Here, we present a brief overview of the DNA based biomarkers described in neuroblastoma and briefly touch upon the standardized operating procedures (SOPs) formalized by the biology subcommittee of the International Neuroblastoma Risk Group (INRG) for performing neuroblastoma tumor molecular diagnostics (15).

Moreover, a recent paper by Pugh and co-workers, summarizing a large number of sequence data, indicates a low median exonic mutation frequency and only few recurrently mutated genes. Among these, *ALK* was most frequently affected (9.2%); all other genes were found to be mutated in less than 3%: *PTPN11*, 2.9%; *ATRX*, 2.5%; *MYCN*, 1.7%; *NRAS*, 0.83% (16). This study was completed by the findings of *ARID1A* and *ARID1B* mutations/deletion in 11% of analyzed tumors (17).

In tune with current development, we will focus mainly on the use of an ultra-high density (UHD) single nucleotide polymorphism array (SNParray) technique for the molecular diagnostic work up of neuroblastic tumors. So far only very few studies applied SNParray techniques in the diagnostic work up of neuroblastomas. The first reports on SNP array data of neuroblastoma tumors were done by an American and a British group both in 2007 (18, 19). George and co-workers described a high concordance between 1p, 3p, and 11q deletion and LOH and reported on copy neutral LOH (cnLOH) at the short arm of chromosomal arm 11p. The Newcastle group correlated SNP array data with expression data. In 2011, Kryh and co-workers using a SNP array, found cnLOH as a frequent event in neuroblastoma cell lines (20). Souzaki et al. reported in their SNP array study on a significantly higher number of SCAs in older patients and a possible correlation of the number of SCAs and outcome in diploid/tetraploid neuroblastomas without MNA, however no, information on LOH data was given (21).

To learn about the applicability of an UHD genome-wide SNP array technology in the diagnostic work up of neuroblastomas, we introduced a SNP array platform (CytoScan^®^ HD Array) in our laboratory in 2012. This pan-genomic technique increased the diagnostic yield enormously in comparison to the hitherto limited but nevertheless useful multilocus (neuroblastoma-specific) MLPA technique and the single loci interphase FISH (I-FISH) investigations. The latter, in fact, will probably not become obsolete owing to its very specific advantages, i.e., the analysis of single cells, the possibility to investigate tumor samples with low tumor cell contents and with intratumoral heterogeneities, especially in case of oncogene amplifications. In addition, I-FISH results provide information on the somy level of the target chromosome, thus help to define the base line in array studies of aneuploid tumors, especially in complex mosaic situations.

Since 2012, we retrospectively and prospectively worked off a large number of neuroblastoma samples and performed SNP data analysis and interpretation by the ChAS software or, for comparison of larger number of tumor samples, by the Nexus software. The individual SNP profiles were compared and validated with available FISH and MLPA results.

## Results

### Visualizing the whole tumor genome

**Figure 1:**
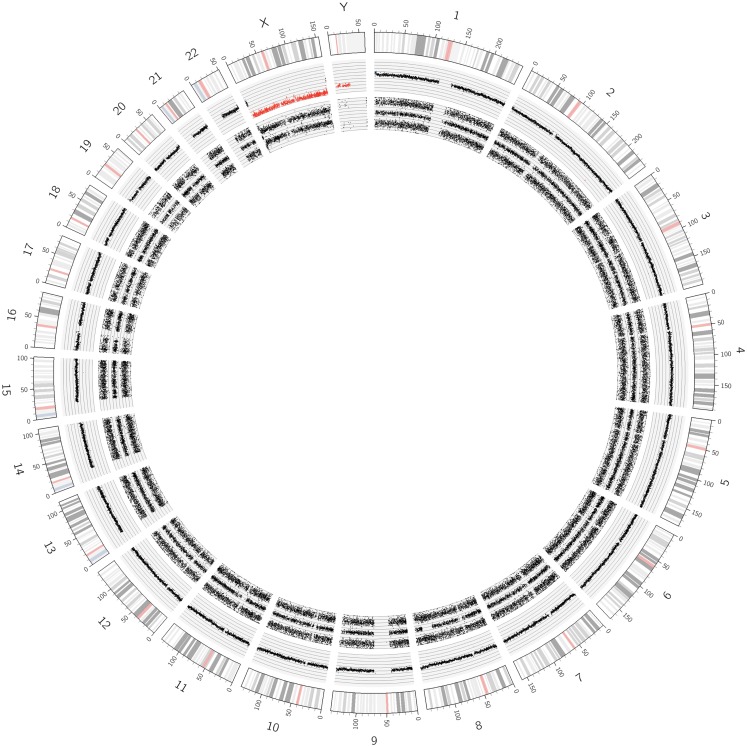
**Circos plot showing the normal chromosomal complement from a Schwann cell dominant tumor**. The outer ring indicates the chromosome ideograms and distances in Mb. The next ring indicates the copy number information. All autosomes are disomic (black dots), the X and Y chromosomes are present in one copy each (red dots). The innermost ring represents the allele peak frequency of the chromosomes. All autosomes disclose three allele peak tracks whereas the X and Y chromosomes show only two tracks; exception: the pseudo-autosomal regions on the tips of the X chromosome, which have three allele tracks.

**Figure 2:**
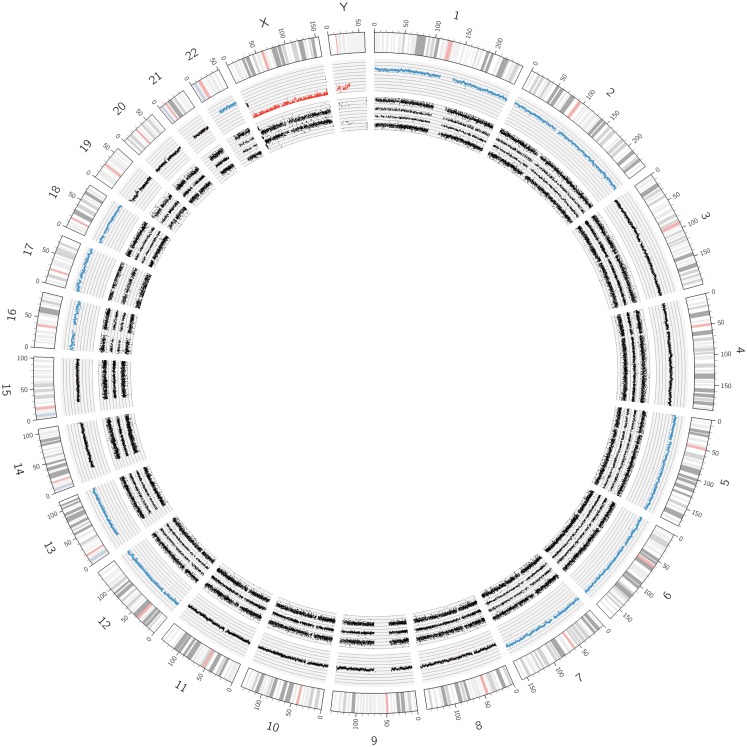
**Circos plot of a near-triploid neuroblastoma**. Chromosomes 1, 2, 5, 6, 7, 12, 13, 16, 18, and 22 are trisomic, represented by the blue dots in the copy number track and four tracks each for the allele peaks. Note that chromosome 17, despite showing three allele peak tracks, is not disomic but tetrasomic, as can be seen by the blue copy number dots. All other autosomes are disomic.

Although in the daily routine work, the single chromosome genome analysis – as it is done, e.g., by the ChAS software (for details see Materials and Methods section and Supplementary Materials) – is indispensable, we start our introduction into UHD SNP array analysis with a whole genome visualization. One way to present all genomic changes in one graph is to use the so called Circos plots. This type of visualization tool allows the identification of copy number changes at one glance. In the different Circos plots presented in this paper (Figures 1, 2, 6, and 7 and Figure S2 in Supplementary Material), the following information is provided: the chromosome ideograms with the Mb information are located along the outside of the diagram. It is followed inwards by the copy number probe track and the allele frequency track. In case there are whole chromosome/segmental gains or losses, the copy number track is labeled in the usual color code, i.e., blue color indicates gain or amplification of chromosomal material and red indicates loss of material.

**Figure 3:**
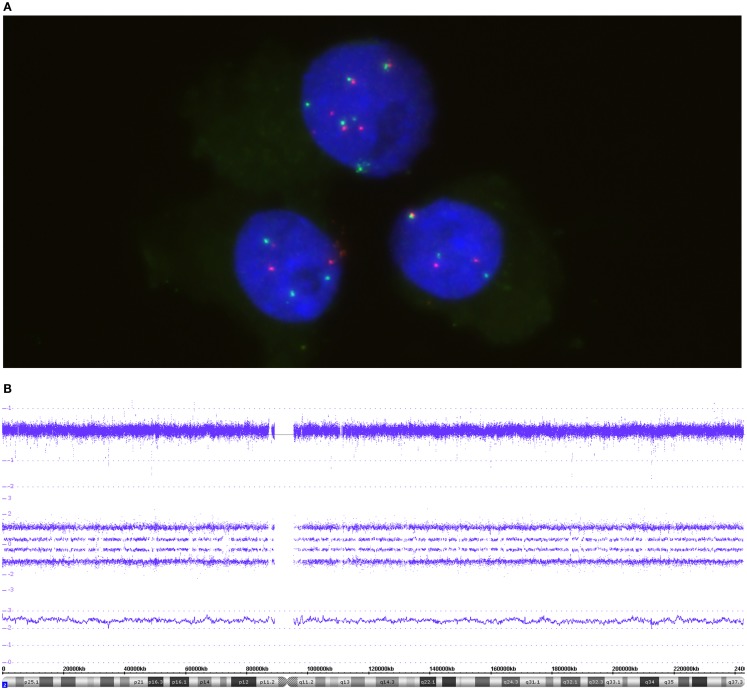
**The FISH picture in (A) highlights three nuclei co-hybridized with *MYCN* and 2p specific probes**. Both probes, *MYCN* (green) and the reference probe (red), are present in a balanced manner – either 3 pairs or 6 pairs in the upper cell. **(B)** The SNP array profile for chromosome 2. Copy number probe intensities (log_2_ ratio) are represented in the upper track. The four allele peak tracks in the middle indicate a trisomic situation due to the allele distribution AAA, AAB, ABB, and BBB. The smooth signal above the ideogram is between 2 and 3 because of difficulties in base line adjustment in cases of triploid tumors.

In Figure 1, SNParray results from normal cells, which occur and expand during the maturation process of neuroblastoma, i.e., from the ganglioneuroblastoma/ganglioneuroma-associated Schwann cells, are presented in a Circos plot. All autosomes clearly show three single tracks in the allele peak circle as it is expected for normal cells. The allele peak tracks consist of single points, each of which representing a single SNP interrogated by “A” and “B” allele probes. Thus, the three tracks reflect the allele combination AA, AB, and BB, which is in line, together with the copy number information, with a disomy of all chromosomes with the exception of the X and the Y chromosomes, which are present in one copy each, reflected by only two allele peak tracks (allele distribution: AA, BB) and the red labeled copy number tracks. In contrast to the neuroblastoma-associated Schwann cells, neuroblastoma cells with differentiation capacity and with ganglionic differentiation usually show numeric (whole) chromosome aberrations as given in the Circos plot of Figure 2 and the FISH picture and the single chromosome view in Figure 3.

**Figure 4:**
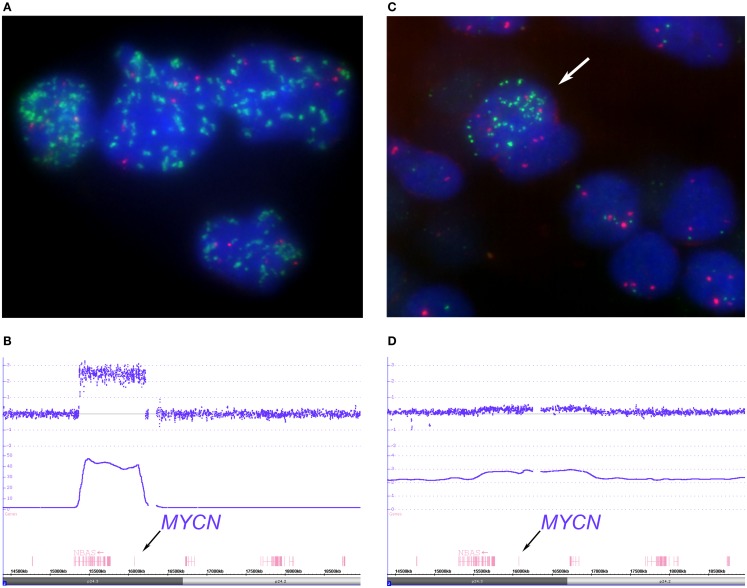
**Depicts examples of homogenous “classical” and heterogeneous *MYCN* amplified tumors**. The FISH picture, **(A)** (*MYCN* FISH probe in green, 2p probe in red), shows tumor cell nuclei with a varying number of *MYCN* signals ranging from approximately 30 signals up to hundreds distributed randomly within the nuclei. The large size of some of the *MYCN* hybridization spots can be explained by repeated amplicon units. The SNParray profile of a segment of the short arm of chromosome 2 from a homogeneously amplified neuroblastoma is given in **(B)** which shows a clear peak for the *MYCN* locus (copy number ~48). **(C,D)** Examples of a so called hetMNA tumor. In the I-FISH picture **(C)**, one tumor cell nucleus clearly displays *MYCN* amplification (arrow), while the others do have a balanced number of *MYCN* and reference probe hybridization signals. The SNParray profile **(D)** shows an example of a heterogeneous *MYCN* amplified tumor with much lower peaks (copy number < 3 of the smooth signals, lower line) than compared to the profile given in **(B)** and could easily be missed if the number of tumor cell nuclei with *MYCN* amplification is too small in the sample under examination.

**Figure 5:**
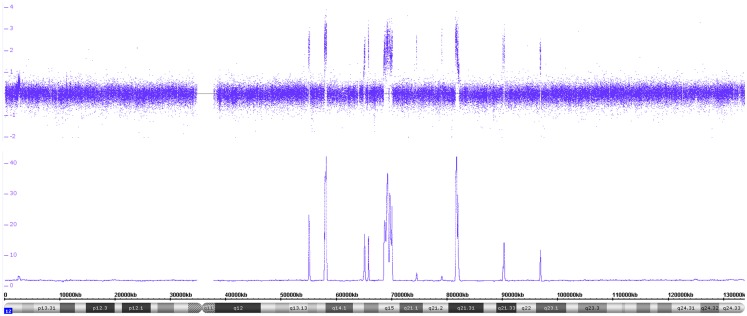
**The copy number data disclose a high number of amplicons on the long arm of chromosome 12**. The log_2_ copy number track and the smooth signal track show a number of copy number peaks ranging from ~10 to ~42 copies. The different amplicons contain the following genes *MDM2, MDM1, CDK4, DCD, INHBC, GLI1, OS9, METTL1, RSSFJ, GNS, MSRB3*, and *IL26* besides a number of other ones.

Due to the design of a Circos plot, only a condensed form of data can be presented. For detailed analyses, as shown in Figures 3–5 and 8–10 (and Figure S1 in Supplementary Material), SNParray data are given in single chromosome (complete or detail) views. The data in this detailed graphical visualization according to the ChAS software are arranged differently as compared to the Circos plot. The cytoband information at the bottom of the graph is followed by the gene annotations (red bars) in the next track. In this lower field of the detailed view, the software also allows for the visualization of information which is not shown in this presentation. Most importantly, the copy number variations (CNVs) with the corresponding annotations can be shown for every genomic locus, thus helping to better interpret microdeletions and -gains (Figure S1 in Supplementary Material). The next track in Figures 3–5, 8 and 10, represented by a line, indicates the so called “smooth signal” – the underlying algorithm performs a smoothing of the copy number information thus providing helpful, albeit sometimes crude, information on the copy number state of the chromosome or parts thereof. This information in combination with the log_2_ information and, importantly, together with the allele frequency data, allows a detailed identification of the underlying genomic changes.

The visualization of the whole genome from a near-triploid tumor presented in form of a Circos plot is shown in Figure 2. Ten autosomes show gains of whole chromosomes resulting in a trisomic state, chromosome 17 is tetrasomic and all other autosomes are disomic. In Figure 3B, a close up single chromosome view shows a trisomy 2. The whole chromosome gain is discernible by a copy number state above 2.0 (Figure 3B, see smooth signal; concerning “baseline problems” see below and figure legend) and, importantly, also by the allele frequency which indicates the exact somy status. The allele track (the middle track in Figure 3B) shows four single tracks representing the “allele distribution” AAA, AAB, BBA, and BBB which is in line with a trisomy. The equal spacing between the tracks points to a pure trisomic population, thus indicates a baseline “error” (see below). A FISH image of differentiating neuroblast nuclei is shown in Figure 3A applying chromosome 2 subtelomeric and a *MYCN* specific probe. A trisomy 2 is reflected by the hybridization pattern with three pairs of signals per nucleus in the majority of nuclei. Occasional nuclei with a hexasomy 2, as shown in the picture, are not uncommon.

### “High” copy number aberrations – amplifications

Interphase FISH used for the detection of gene amplifications can be considered as classical diagnostic tool for such purposes, as it is a quick and reliable technique and allows analyses at the single cell level. As seen in Figure 4A, the number of FISH spots varies markedly from cell to cell, ranging from hundreds per nucleus to a small excess of signals in comparison to the reference probe (in red) located on the short arm of chromosome 2. This variation is caused by the random distribution of double minute chromosomes (dmin) to the daughter cells during cell divisions. Despite this unequal dmin distribution among the different cells, we call this a homogeneously amplified tumor, since virtually all tumor cells show supernumerary *MYCN* signals, with a mostly more than four-fold increase in the *MYCN* signal number compared to the reference probe (thus being well above the threshold value as it was defined for neuroblastoma diagnostics). Array based techniques, as well, show distinct peaks, surpassing by far the four times ratio of the fluorescence intensities.

The detailed SNParray data from one *MYCN* amplified tumor are visualized in Figure 4B. The signal intensities of the different genes that are contained in the amplicons (i.e., an amplification unit that can consist of one or more genes) reach copy number values of up to ^~^48. The amplicon contains the full lengths of the *MYCN* gene, the *DDX1*, and part of the *NBAS* gene.

The complete genomic picture of a *MYCN* amplified tumor as presented in Figure 4A is visualized in the Circos plots shown in Figure S2 in Supplementary Material. The Circos plot shows only very few SCAs, i.e., loss of 1p and gain of 17q.

Besides the more frequently found “classical,” i.e., homogeneous, *MYCN* amplification, neuroblastomas can also show intratumoral heterogeneity for the *MYCN* status, clearly detectable by the use of FISH techniques, which allow the identification of single cells. In these tumors, various proportions of tumor cells with amplification (and gain) of this gene and tumor cells without this aberration – yet unambiguously identifiable as tumor cells – coexist (Figure 4C). For identifying intratumoral heterogeneity of *MYCN* amplification, FISH is the method of choice, since routinely applied array techniques are based on the DNA extracted from a large number of cells and not on the visualization of single cells. The amplification will be visible only if the number of amplified cells in the sample and the degree of amplification surpasses a certain threshold. This holds also true for the UHD SNParray even if its sensitivity is very high. If a subclone of amplified cells is detected, the amplicon is mostly only visible as a gain. A ChAS generated image of a tumor area with only a minority of tumor cells disclosing the amplification is shown in Figure 4D. The log_2_ ratios of this SNParray sample correspond to roughly three copies. The SNParray pattern of a gain of the short arm of chromosome 2 or of parts thereof differs from the pattern of a heterogeneous amplification regarding the size of the gained region, which markedly exceeds the amplicon size, i.e., the *MYCN* gene and its adjacent genes or loci.

However, in neuroblastoma tumors, not only *MYCN* and adjacent genes can be affected by an amplification process, but also *ALK* (see below and Discussion section) and other genes/loci on chromosome 2 (not shown). Furthermore, the chromosome 12q arm contains other – in neuroblastoma not infrequently amplified – genes, among them oncogenes and cell cycle-associated genes (*MDM2, CDK4, OS9*, and *GLI1*, see Figure 5).

### “Low” copy number aberrations – segmental gains and losses

**Figure 6:**
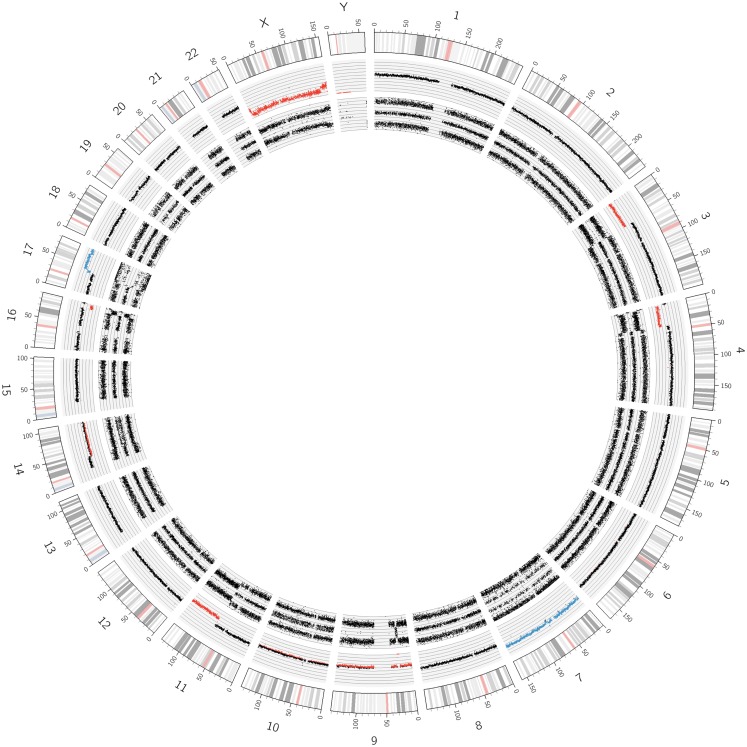
**A Circos plot of chromosome 11q deleted neuroblastoma**. The red track in the log_2_ copy number lane already indicates the loss of genomic material. The two allele peak tracks indicate LOH. In addition, the short arms of chromosomes 3 and 4 and the long arm of chromosome 16 show deletions accompanied by LOHes. The chromosome 9 shows a whole chromosome UPD and a 9p21.3 loss resulting in a nullisomy for the genes *CDKN2A* and *2B*. Further segmental aberrations were found on chromosomes 14 and 17. A whole chromosome gain was found for chromosome 7.

Segmental chromosomal aberrations, defined as gains or losses of larger chromosomal regions, are hallmarks of most aggressive neuroblastomas. Losses of total chromosomal arms or parts thereof are frequently associated with loss of heterozygosity (LOH) of the affected chromosomal region. Chromosome arms 1p and 11q are most frequently involved in these processes (Figure 6 and Figure S2 in Supplementary Material). These two aberrations are part of seven recurrent segmental gains and losses currently regarded as prognostically meaningful or “‘typical” SCAs (i.e., losses at 1p, 3p, 4p, and 11q and gains at 1q, 2p, and 17q). All other segmental aberrations are presently called atypical SCAs. The Circos plot in Figure 6 shows a typical deletion at the long arm of chromosome 11 (11q13.4-qter). The copy number changes from two copies to one copy is represented by the red labeled dots and is accompanied by the loss of allelic probes resulting in the appearance of only two allele probe tracks reflecting the A and B status of this region. The non-affected part of the chromosome pair shows both the normal copy number pattern and the typical three allele tracks. Especially in mosaic situations, the additional allele information can be of great help to unambiguously identify copy number changes.

### Multiple segmental gains and/or losses within confined regions of the genome – chromothripsis

**Figure 7:**
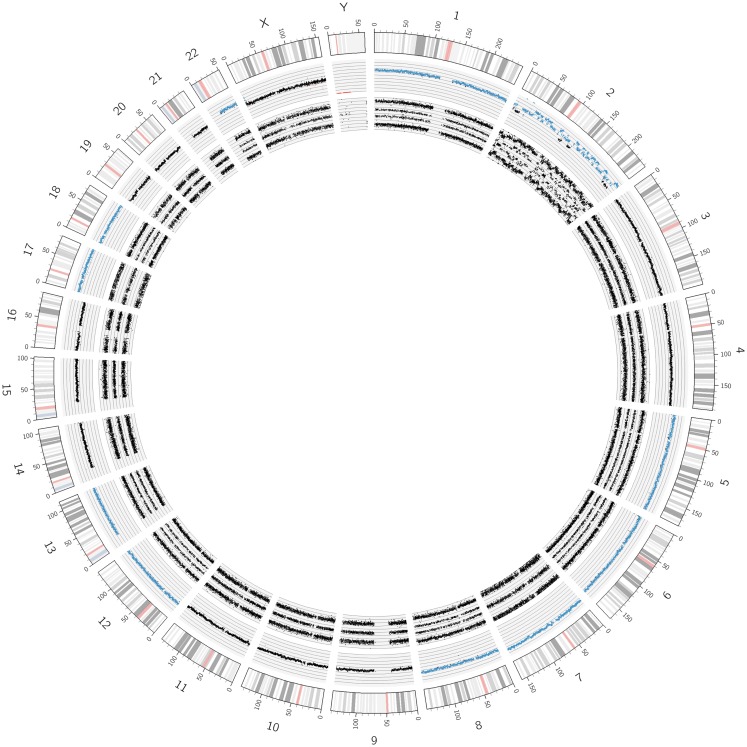
**An example of the recently described “chromothripsis**.” As visualized by the Circos plot, only chromosome 2 but no other chromosome showed structural aberrations. However, the concerned chromosome underwent extreme shattering and reshuffling, as detailed in Figure 8.

**Figure 8:**
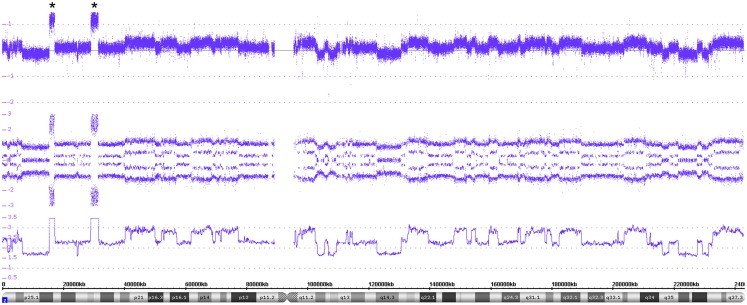
**A detailed view of the genomic features of the chromothripsis case described in Figure 7**. The chromothripsis with 60 breakpoints lying in both chromosomal arms (chromosome 2) lead to a large number of copy number variations represented in an oscillating copy number profile (visible in all tracks). In addition, the profile shows two amplicons including *MYCN* and *ALK* besides other genes (highlighted by asterisks).

An example of chromothripsis affecting the whole chromosome 2 is shown in Figures 7 and 8. The Circos plot of this tumor already indicates a large number of aberrations in this chromosome and the detailed view done with the ChAS software allows a precise annotation of the breakpoints along the chromosome. In addition, this tumor shows a *MYCN* as well as an *ALK* amplification (Figure 8). In case of chromothripsis, the copy number changes typically oscillate between gains or losses of one and two copies visible in the copy number state and the SNP profile. So far, we have detected chromothripsis in 4.3% of neuroblastomas (10 out of 233 evaluable tumors, all stages). These patients with chromothripsis tumors had predominantly high stage tumors. In three tumors, this aberration was associated with amplification of the *MYCN* gene, one had a heterogeneous MNA and the others were not amplified.

### Alterations of single genes – intragenic gains and losses

**Figure 9:**
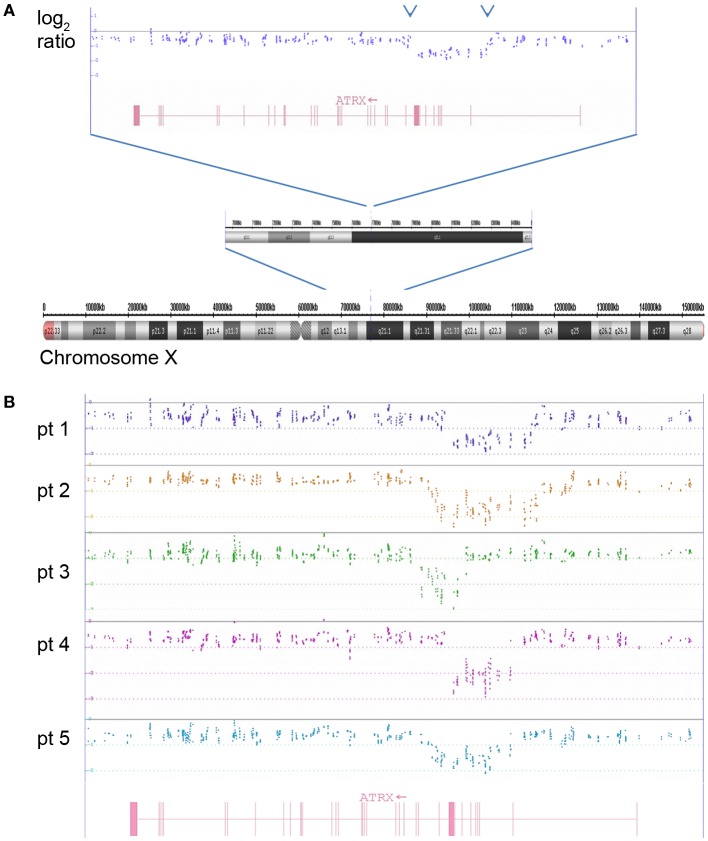
**The log_2_ copy number plot displays the structural variations in the *ATRX* gene**. **(A)** Complete chromosome X with small deletion at Xq21.1 visible in the zoomed-in chromosomal sub-band. The dots indicate the log_2_ copy values of individual copy number probes. The intron–exon structure of *ATRX* is shown in red (vertical bars are exons). **(B)** Five examples of *ATRX* deletions spanning different parts of the gene are depicted (log_2_ ratio from five neuroblastomas are shown in different colors analyzed with the ChAS software).

Figure 9 shows the X chromosome with a close up highlighting the *ATRX* gene located in the cytoband Xq21.1. In the upper part of the Figure 9, the copy number probes show a clear deletion spanning from exon 2 to exon 8. In the lower part of the figure, five examples of *ATRX* gene deletions are given. Among the 213 evaluable samples analyzed in this study, an intragenic deletion of the *ATRX* gene was found in 21 tumors (9.9%). All *ATRX* deleted tumors also showed the ALT phenotype (alternative lengthening of telomeres) that is typical for the loss of the *ATRX* gene function (data not shown).

### Copy neutral variations – uniparental whole chromosome and segmental LOHes

**Figure 10:**
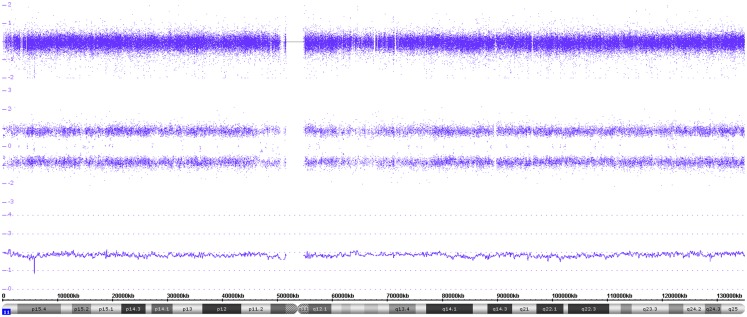
**Gives an example of a whole chromosome uniparental isodisomy**. Both the log_2_ copy values and the smooth signal indicate two copies of chromosome 11. However, only two allele peak tracks are visible representing the AA and BB alleles.

In case of deletions of whole chromosomes or parts thereof, which are associated with LOHes, a two track SNP probe pattern is visible as shown in Figure 6. However, the typical LOH SNP pattern is not only found in combination with copy number changes but can also occur without losses, i.e., in copy neutral situations. In case the LOHes concern a whole chromosome, they are called UPD (uniparental isotrisomies and isotetrasomies can also occur). In case of segmental UPDs, only parts of a chromosome are concerned (also called: ROH, runs of homozygosity or LCSH, long contiguous stretches of homozygosity). Figure 10 shows a whole chromosome UPD of chromosome 11 in a L2 neuroblastoma without MNA. These aberrations, especially the segmental UPDs can either be constitutional or somatic, i.e., tumor specific. Clarification by analysis of reference DNA is frequently necessary for a final assignment of the aberration to the exact category.

## Discussion

### Chances and limits of single nucleotide polymorphism probe analyses used in tumor diagnostics

In cancer research, the great value of genome-wide SNP arrays is constituted by the combined information of copy number changes and allele status by SNPs in pure and mosaic situations. The Affymetrix UHD SNP array (CytoScan^®^ HD Array) contains more than 1.9 million non-polymorphic markers and over 740,000 SNP markers with an average intragenic marker spacing of 880 bps and intergenic marker spacing of 1737 bps. The high non-polymorphic marker coverage guarantees the reliable detection of copy number changes (variations and alterations, see below) even in the range of microdeletions or microgains, i.e., small deletions/gains in non-coding regions/genes and even in single introns or exons (dependent on the coverage of the concerned region, see for example the *ATRX* gene section and Figure 9). The combination of copy number with the allele information, i.e., the zygosity status, enables the detection of LOHes with copy losses as well as copy neutral allelic changes. In addition, the UHD SNP array has a high sensitivity for mosaicism detection, which, in the literature, is associated with a detection limit for mosaicism of less than 5% (22). The limits of the technique concern translocations. In case they are balanced, without gains or losses of sequences, they are not detectable by this technique. If gains or losses occur next to known translocation breakpoints, the presence of such alterations, i.e., gene rearrangements, can be deduced indirectly in a portion of rearranged tumors (23). The only technique which can overcome this problem, also in cases of unknown or unexpected translocations, is whole genome sequencing. FISH is helpful and of diagnostic value only in cases of known and suspected translocations for which FISH probes exist. In neuroblastoma, however, balanced translocations are described to represent only very infrequent events.

A so far unsolved software problem concerns the normalization of the copy number information, i.e., the calculation of the “baseline.” In case of aneuploid DNA contents, the ChAS (as well as the Nexus) software is often not able to calculate the correct monosomic or disomic levels, i.e., the “baseline.” This is, however, crucial in order to discriminate, e.g., odd number of (whole or segmental) chromosomal gains from mosaics with LOHes, which both end up with four allele tracks. After correct baseline assessment, “absolute” chromosome numbers can easily be determined.

### Individual variants – the copy number variations

Since UHD SNP arrays are able to capture even micro-alterations, we have to turn to the inter-individual variations of human genomes, which – with the introduction of microarray-based techniques ten years ago – also came to the knowledge of scientists in oncology research. These variations have to be considered when investigating tumor genomes by SNParray techniques in order not to mix-up germline CNVs with tumor-specific aberrations, i.e., copy number alterations (CNAs). CNVs are deletions or duplications in the genome, occurring in non-coding as well as in coding regions, which can vary considerably in size, ranging from 50 base pairs to 3 megabases. The causing events include non-allelic homologous recombination, non-homologous end-joining, transposition of transposable elements or pseudogenes, and variable number of tandem repeats or replication errors (24). The prevalence of CNVs in the human genome is estimated to amount to 0.4% (25). Recent studies suggest that the average human genome contains >1000 CNVs, covering approximately four million base pairs (26, 27). SNPs variations, on the other hand, are supposed to account for approximately 0.1% of human genome inter-variability. Consequences of CNVs include gene dose effects, with reduced or increased protein expression, and also truncated protein sequences. While most common CNVs have little or no role in causing diseases (28), some have been associated with susceptibility or resistance to diseases (29).

In case small gains or deletions are present in a tumor-derived SNP profile, the database of genomic variation (DGV), which currently contains over 100.000 published CNVs in the human genome (24), can be helpful in the assignment of the variation/alteration. However, if an unequivocal assessment of the copy number changes as either common variants or as tumor-specific is necessary, analysis of constitutional DNA is imperative. Furthermore, critical issues may arise if a change is clearly identified as a disease-associated CNV (constitutional) [the issue of reporting and integrating CNV identification in medical reports is addressed by the electronic medical record and genomics (eMERGE) consortium (24)].

### Loss of heterozygosity and uniparental isodisomies

So far, FISH, MLPA, and aCGH have been the main tools in neuroblastoma diagnosis. Since these techniques give only information on copy number changes, i.e., gains and losses, without the possibility to infer the allele status (the only exception being the detection of a monosomy, which definitely indicates the presence of an LOH), cnLOHes, which may concern chromosomal segments or whole chromosomes, have been beyond diagnostic possibilities and therefore generally neglected in neuroblastoma research/diagnosis.

PCR-based LOH techniques, on the other hand, have only been applied for some selected chromosomal regions (e.g., 1p36.33) leading to sometimes misleading results due to, e.g., constitutional segmental UPD. Thus, one of the merits of SNP arrays is the detection of acquired (tumor-specific) cnLOHes whose frequency has always been underestimated. Acquired whole chromosome uniparental somies reflect the presence of identical chromosomes, which are not necessarily disomic, but occur also, especially in neuroblastoma, in the case of trisomies and tetrasomies (theoretically, all possible somy numbers can occur in combination with a total LOH).

Originally, the term “uniparental disomy” was introduced by Engel in 1980 (30) in the context of atypical diploid zygotes. According to his concept, they can arise from the fusion of nullisomic with normal monosomic gametes (with subsequent monosomy rescue) or monosomic with disomic gametes (with subsequent trisomy rescue). This kind of (inherited, not acquired somatic) uniparental derivations of a certain chromosome can then either be isodisomic (arisen from monosomy rescue, i.e., duplication) or heterodisomic (by trisomy rescue with loss of the sole chromosome from the other parent). The latter uniparental and heterodisomic situation would not be recognizable by the SNP profile.

In case of whole chromosome somatic uniparental somies, which are acquired during tumor evolution or progression, we are always dealing with “isosomies,” which will be at the disomic, trisomic, or other somy levels (isodisomies, isotrisomies, and isotetrasomies). The identity of the chromosomes is clearly recognizable by the typical SNP profile reflecting LOH, i.e., the presence of only two allele tracks. Mitotic errors with subsequent monosomy or trisomy rescues can be regarded as causative for this kind of aberration. LCSH or ROH show the same SNP profile pattern as described above, but are restricted to chromosomal segments. They arise from somatic/mitotic recombinations (homologous recombination) or double strand break repair and are not necessarily acquired but can also be present constitutionally. If less than 3 Mb in size, such LCSH are found in most individuals and may total up to 2–5% of the genome (31). Even larger and multiple LCSH up to 15Mbs are not necessarily tumor specific but may arise from consanguinity. Thus, as already mentioned in the context of small copy number changes, the investigation of constitutional DNA is often necessary to clarify this issue.

### Whole chromosome aberrations – tumor cell ploidy and new techniques to determine an “old prognostic indicator” in neuroblastoma

In neuroblastoma, evaluation of the tumor cell content is a long-standing tradition (32). Due to its prognostic impact – two genetic subtypes based on differences in the ploidy levels, which are associated with age and prognosis as well – ploidy was quickly implemented into risk-grouping schemes (e.g., INRG). On the whole, a hyperdiploid (near-triploid or -penta-/-hexaploid) DNA content slightly prevails (32, 33). Locoregional neuroblastomas are commonly hyperdiploid. However, in ganglioneuroblastomas and ganglioneuromas and possibly also in differentiating neuroblastomas, the large amount of diploid Schwann cells can mask the aneuploidy of the neuroblastoma cell population (see “UHD SNP array results and pitfalls in case of spontaneous neuroblastoma maturation and regression” section).

While exact DNA values can only be generated by FACS measurements, SNP arrays give the somy numbers of all chromosomes from which the DNA content can be deduced, provided the baseline (see above) was correctly assigned to the disomic level. The adapted Circos plot tool as described by Krzywinski et al. (34) is a helpful tool to visualize these whole chromosome aberrations (Figure 2).

Besides diploidy (di-tetraploidy), an aneuploid (near-triploid, -pentaploid, -hexaploid) DNA content is commonly encountered (15). The somy levels of the single chromosomes can vary from di- to multisomic. Monosomies, however, are virtually never found in neuroblastomas. Although near-triploid neuroblastomas have been considered as almost consistently favorable tumors, they can, as diploid neuroblastomas usually do, show segmental aberrations and even oncogene amplifications. Original tumor ploidy evaluations were based on classical cytogenetic approaches or flow cytometric measurements. However, chromosome studies, though potentially very informative, have different disadvantages and therefore are not used in the clinical routine anymore. Flow cytometry or static photometry are excellent tools for this purpose, but cannot provide information on structural chromosome aberrations of the tumor cell population. Today, whole chromosome gains or losses leading to changes in the ploidy level can elegantly be recorded by pan-genomic techniques (CGH and SNParray) with the great advantage that also information on structural aberrations, i.e., amplifications or typical or atypical SCAs can be obtained simultaneously.

A number of studies have shown that in patients less than 18 months of age with metastatic disease, hyperdiploidy in combination with a non-amplified *MYCN* gene, and a lack of specific SCAs (such as 11q deletion) is commonly predictive of a favorable outcome (10, 11, 35). However, despite the fact that tumor cell hyperdiploidy (usually near-triploid DNA content) is frequently associated with spontaneous maturation (36) or spontaneous regression (14, 35), this ploidy level alone cannot guarantee a favorable tumor behavior. The long-standing view, that tumors with this ploidy level almost always lack MNA and SCA, is a concept that cannot be maintained anymore. It is estimated that at least 20% of hyperdiploid tumors in localized stages and the vast majority in metastatic stages (except for stage MS) contain structural chromosome rearrangements, including MNA (Ambros, personal observation), which lead to a clinically aggressive tumor behavior (13, 14). As already mentioned above, the somy- (copy number of individual chromosomes) and ploidy- (number of sets of chromosomes in the nucleus) level can also be depicted on the basis of SNParray data by a combined analysis of the copy number and SNP data.

### Gene amplifications

Neuroblastomas with an unfavorable/aggressive clinical behavior have a high tendency for locally invasive growth and widespread metastatic dissemination via the lymphatic and hematogenous systems. Development of resistance to cytotoxic drugs during the course of the disease is an important reason for treatment failure. High-level amplification of the *MYCN* gene – besides a few other amplified genes – is detected in 20–25% of neuroblastomas. *MYCN* amplification has been shown to be strongly associated with rapid tumor progression and poor prognosis in patients of all ages, independent of the stage of disease (with the exception of stage 1) (5, 8, 9, 37). Frequently, adjacently located genes, like *DDX1, NAG* (*NBAS*) or, more rarely, the proximally located *ALK* gene (less frequently involved), are co-amplified with *MYCN* (18). Recent data indicate that information about *ALK* amplification/mutation may be of interest when looking for alternative treatment strategies such as ALK inhibitors (38). In addition, genes located on chromosome 12q, like *MDM2* and *CDK4* (which may be amplified in the presence or absence of the *MYCN* gene), may indicate a subset of tumors with a unique clinical behavior (own observation; Martinsson, personal communication).

The occurrence of intratumor heterogeneity of MNA (hetMNA) has been reported by different groups (39–41). In the SNP array profile, the intensity peaks are less high when only a sub-population of the cells carries the amplification, as exemplified in the hetMNA tumor presented in here. Caveat: array data *per se* are usually not sufficient to discriminate hetMNA from homMNA. Low *MYCN* peaks can also be due to a high normal cell content (e.g., after chemotherapy or caused by sampling error). Therefore, I-FISH *MYCN* data should be generated in any case. All in all, the clinical meaning of hetMNA is still unclear regarding its effect on patients’ assignment to specific treatment strategies. Therefore, a detailed analysis of a large number of hetMNA tumors and a link with the clinical data is crucial. Although the presence of homogenous amplification of the *MYCN* oncogene is central to the risk stratification systems in virtually all cooperative clinical trial groups, it is important to emphasize that the majority of aggressively behaving neuroblastomas do not show amplification of this oncogene. Thus, other genomic features are associated with/responsible for aggressively growing tumors. In the following, we describe the currently best-known genomic changes that are associated with a clinically unfavorable behavior.

### Segmental chromosomal aberrations

SCAs, especially as the “typical” ones described above, have repeatedly been shown to be present in tumors with unfavorable behavior. They manifest as losses or gains of larger chromosomal segments spanning from at least 3 Mb (by definition) to a complete arm of a chromosome. Historically, deletions at the short arm of chromosome 1, especially of the chromosomal region 1p36.3, were among the first genomic aberrations described in neuroblastoma (33, 42). However, as this aberration is frequently associated with MNA, it lost importance as prognostic marker. Conversely, the deletion at the long arm of chromosome 11 (11q) has gained importance over the last years, since these deletions occur predominantly in tumors without *MYCN* amplification. The 11q deletion has thus emerged as a powerful biomarker of outcome in addition to *MYCN* amplification (43–45). Another prominent and very frequently observed SCA, is the unbalanced gain of the long arm of chromosome 17 (46–48). In addition, some studies have shown that deletions at the chromosomal regions 3p, 4p, 9p, and 12p, which do occur at low frequency, can have prognostic impact (35, 49, 50). Recent publications clearly demonstrate the potential of genome-wide approaches to further refine the prognostic accuracy of somatically acquired chromosomal alterations (18, 35, 51–54), and numerous studies have demonstrated that in tumors without MNA, SCAs are frequently associated with clinically aggressive disease – for a summary see, e.g., Ref. (12). However, as already briefly discussed, the influence of SCAs on tumor behavior can differ in different age groups. The genomic data from patients with localized, resectable, tumors without MNA not receiving cytotoxic treatment and enrolled in the LNESG 1 trial (Localized Neuroblastoma European Study Group 94.01) showed a strong prognostic impact of SCAs in patients over 18 months at diagnosis which could not be observed in younger patients (Ambros, personal observation).

### From the detection of chromothripsis to gene deletions

All described aberrations presented below – from chromothripsis to gene deletions, like *ATRX, ARID1A* and *ARID1B* genes – have, so far, only been described by sequencing methods of the neuroblastoma genome. They can, however, also be detected by UHD SNParray.

#### Chromothripsis

The recently described phenomenon named “chromothripsis” represents a shredding of single chromosomes or parts thereof and subsequent random reassembly of the fragments. Molenaar and co-workers describe this catastrophic event, which is significantly associated with unfavorable prognosis, in 18% of high-stage neuroblastoma tumors (55). The breakpoints occurring in this aberration can affect genes known to be involved in translocation processes and, in addition, a chromothripsis chromosome can also bear amplified regions.

According to the definition given by Korbel and Campbell, the CNV between affected and non-affected chromosomal regions is mostly limited to gains or losses of one copy (56). Even though most studies describing chromothripsis applied whole genome sequencing techniques, the use of the cost effective UHD SNParray identified this peculiar genomic aberration without doubt.

#### *ATRX* gene

A detailed study by Cheung and co-workers showed that mutations affecting the X-chromosome-located *ATRX* gene were predominantly found in tumors from older children and adolescents, but less frequently in younger children and not at all in tumors from infants (0–18 months) (57). Mutations of the *ATRX* gene are associated with X-linked alpha thalassemia and mental retardation and encode a subunit of the chromatin-remodeling complex involved in H3.3 incorporation into heterochromatin at centromeric and telomeric regions (58, 59). Similar to the study of Cheung and co-workers, this aberration was exclusively found in patients older than 18 months (mean age: 6.6 years, median: 4.7 years; range: 2.7–20 years) and was always associated with the ALT phenotype.

#### *ARID1A* and *ARID1B*

Among the genes not previously known to be involved in neuroblastoma, deletions and sequence alterations of the chromatin-remodeling genes *ARID1A* and *ARID1B* were identified in 11% (8 of 71 tumors), and these aberrations correlated with a more aggressive phenotype (17). The SNParray technique used in this study identified deletions affecting ARID1A in only one tumor (data not shown).

### UHD SNP array results and pitfalls in case of spontaneous neuroblastoma maturation and regression

Maturation not induced by cytotoxic therapy leads to the formation of ganglioneuroblastomas (GNB) and ganglioneuromas (GN) and is a prognostically favorable sign (with the exception of a subgroup of nodular GNB). A gain of intact chromosomes resulting in a rise of the DNA content in a near-triploid (-penta-, -hexaploid) range (3, 10, 35, 36, 52) and absence of SCAs are characteristic of the neuroblastic/ganglionic cell population in Schwann cell stroma-rich tumors while the Schwann cell population itself has a diploid DNA content (36, 60). Therefore, we have concluded in previous work that Schwann cells in GN and GNB are non-neoplastic, reactive cells that are attracted by neuroblastoma cells and we have always assumed that Schwann cells play a crucial role in the maturation process by stopping tumor cell growth and inducing differentiation of the neuroblastoma cells (61). In terms of diagnostic measurement of the DNA content of neuroblastomas, it is essential to know about the diploid Schwann cell population in order not to ascribe a diploid DNA content to the neuroblastoma cell population in case of a Schwann cell stroma-rich tumor.

A favorable clinical behavior of neuroblastomas is typically characterized by the capacity to undergo spontaneous regression of a tumor residuum or, occasionally, of the complete tumor in localized disease or even of metastasized tumors such as stage MS (former stage 4S) tumors (5). The latter stage occurs in infants up to the age of 18 months and is a unique form of disseminated disease with the capacity of spontaneous regression in case of favorable biological markers. These tumors almost always show gains of whole chromosomes, mostly without SCAs and no gene amplifications (13, 14).

### Genetic markers currently used in clinical trials

Because of the unique clinical/biological heterogeneity ranging from a favorable behavior to very aggressive or indolent/chronic disease and the association of genomic aberrations with the different clinical manifestations, it soon became clear that to implement genomic information into the decision making process was essential. As mentioned above, the *MYCN* status is used in all current neuroblastoma clinical trials. The COG is using 11q and ploidy information in addition to *MYCN* data to apply the most appropriate therapeutic regimen. In Europe, a unique study, the European Low and Intermediate Risk Neuroblastoma Study (LINES), has recently started, implementing SCA data. In case one or more of the seven typical SCAs are affected (i.e., losses at 1p, 3p, 4p, and 11q and gains at 1q, 2p, and 17q), an upstaging in the treatment decision algorithm will be performed (Schleiermacher G. and the SIOPEN Biology Group). This Europe-wide treatment strategy employs real time online quality control of the genomic data before the data are provided to the treating clinician. Yet to allow all this to happen, certain prerequisites had to be fulfilled. A number of technical and interpretation guidelines have been published by the INRG Biology Group covering the following issues: Tumor sampling/storing/reporting of the *MYCN* gene copy status, including analysis of heterogeneous *MYCN* amplified tumors, and the evaluation and reporting of SCAs (15).

## Conclusion

In neuroblastoma patients, information on the genomic profile of the tumor becomes an indispensable prerequisite for adequate treatment planning. For on-going and future studies, the application or implementation of technologies that provide a comprehensive picture of the tumor cell genome is essential. As shown in this paper, high resolution SNParrays will allow new insights into the genomic composition of tumors. To uncover the relatively few known mutations in neuroblastomas, the combined use of SNParray with, for example, targeted sequencing will be a useful combination. In addition, FISH techniques to rapidly determine the *MYCN* status at the cellular level and enabling the detection of intratumoral *MYCN* heterogeneity are expected to remain in use in the diagnostic work up of neuroblastomas. Moreover, it is anticipated that the rich genomic information on stage M tumors which was published in the last few years will stimulate the use of compounds targeting these recently described alterations. Thus, genomic information is not only helpful for evidence-based assignment of patients to specific treatment groups but will also help to discern different types of stage M patients, enabling biology based treatment options.

### Informed consent

As all patients were enrolled in clinical studies, informed consent was obtained from all of them.

## Materials and Methods

### DNA extraction SNParray analyses

In order to obtain high quality SNParray data, we used DNA extracted from fresh or fresh frozen tumor tissue with at least 30% tumor cell content or bone marrow samples applying the “high salt” extraction technique (62). In case of BM samples, the tumor cell fraction was either enriched by magnetic bead separation applying the antiGD2 antibody or extracted from cytospin slides or BM smears, the direct extraction was done only where the percentage of disseminated tumor cells exceeded 30% (for further details see, Abbasi et al., in submission), The Cytoscan HD (Affymetrix, UK) platform was used in all experiments. This array contains more than 1.9 million non-polymorphic markers and over 740 thousand SNP markers with an average intragenic marker spacing of 880 bps and intergenic marker spacing of 1737 bps. The wet lab procedure and all other steps were done exactly according to the manufacturer’s recommendations. Analysis of the SNParray data was done by the ChAS software (Affymetrix, UK) or for comparison of larger number of samples with the Nexus software (BioDiscovery, Hawthorne, CA, USA).

### FISH

FISH experiments and documentation of the results were done as described previously (63).

### Visualization of the SNP array data applying the ChAS software

The visualization tool of the software used in our institution (ChAS) displays a karyoview screen where whole chromosome and segmental chromosome gains and losses as well as LCSH and LOHes can be highlighted (after selection of individual thresholds). The detailed view contains all tracks with the copy number and allele frequency information for each single chromosome separately. For the copy number probes different presentations can be chosen, i.e., the log_2_ ratio, the weighted log_2_ ratio, the copy number state, and the so called smooth signal (Figures 3–5 and 8–10). The allele information is depicted by the allele peaks. Furthermore, CNVs, copy number gains and losses, LCSHs and LOHes, and the presence of mosaics can be highlighted also in the detailed view (Figure S1 in Supplementary Material). The allele peaks – in contrast to the visualization of the B-allele frequency – allow also the recognition of gains and losses in addition to allele imbalances and copy neutral and deletion-associated homozygosities. In addition, the genome can be analyzed at different levels of resolution with exact assignment of breakpoints and identification of genes included in amplicons.

### Circos plots

Circos plots, as shown in Figures 1, 2, 6, and 7 and Figure S2 in Supplementary Material, were created by extracting LRR and allele peak information from ChAS-preprocessed Affymetrix.cychp-files using Affymetrix Power Tools v1.15.2 and a custom Perl script. Individual LRRs were binned into groups of 100 and the bin average taken as smoothed LRR signal. Allele peaks were transformed to BAFs with the formula (allele_peak – 250)/500 and then trimmed to values between 0 and 1. Both smoothed LRRs and BAFs were then plotted using the Circos software v0.64 (34). LRR values ≥ 0.15 are shown in blue (gain) and LRR values ≤ -0.15 in red (loss).

## Conflict of Interest Statement

Affymetrix has paid for one congress visit in 2013 to Peter F. Ambros. The other co-authors declare that the research was conducted in the absence of any commercial or financial relationships that could be construed as a potential conflict of interest.

## Supplementary Material

The Supplementary Material for this article can be found online at http://www.frontiersin.org/journal/10.3389/fonc.2014.00202/abstract
