# Supplementary material for: Ultra-High Density SNParray in Neuroblastoma Molecular Diagnostics
Source: Front Oncol. 2014 Aug 12;4:202. doi: 10.3389/fonc.2014.00202 (PMC4129917; doi:10.3389/fonc.2014.00202)
Supplement: Supplementary file 1 [file Presentation1.PDF]

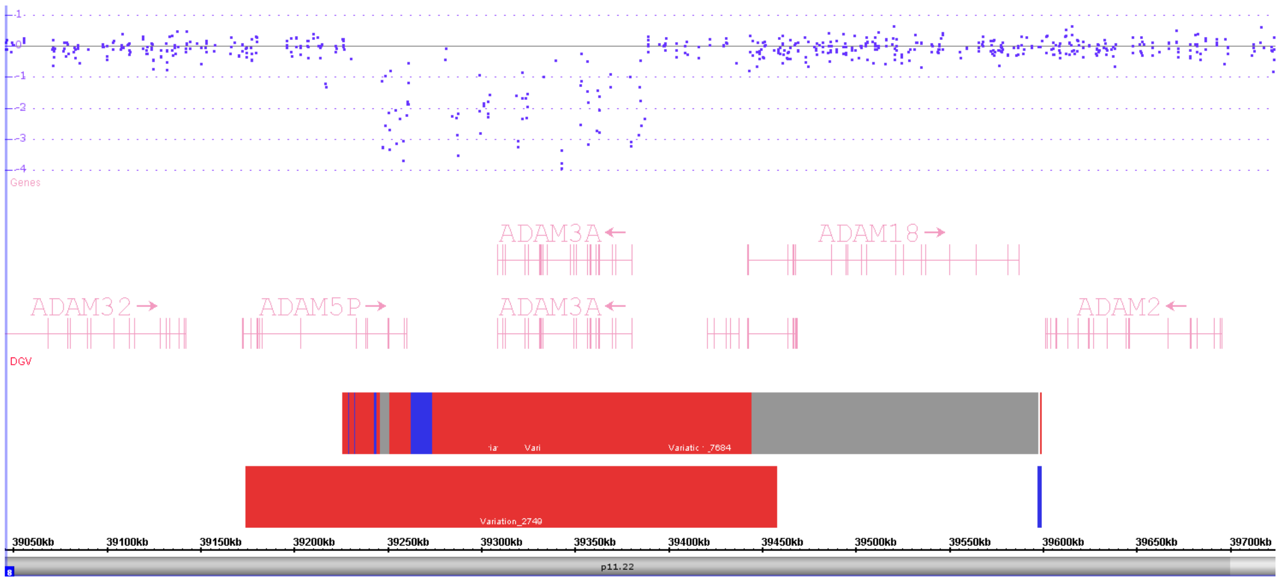

Supplementary Figure 1 - Visualization of a frequently involved CNV region by the ChAS software. The log2 copy number signals show a homozygous deletion in 8p11.22 involving the ADAM3A gene. Besides the copy number information and the SNP information (not shown) also the gene names plus exon information is displayed. In the underlying lane the CNV information are provided allowing to link to appropriate databases.

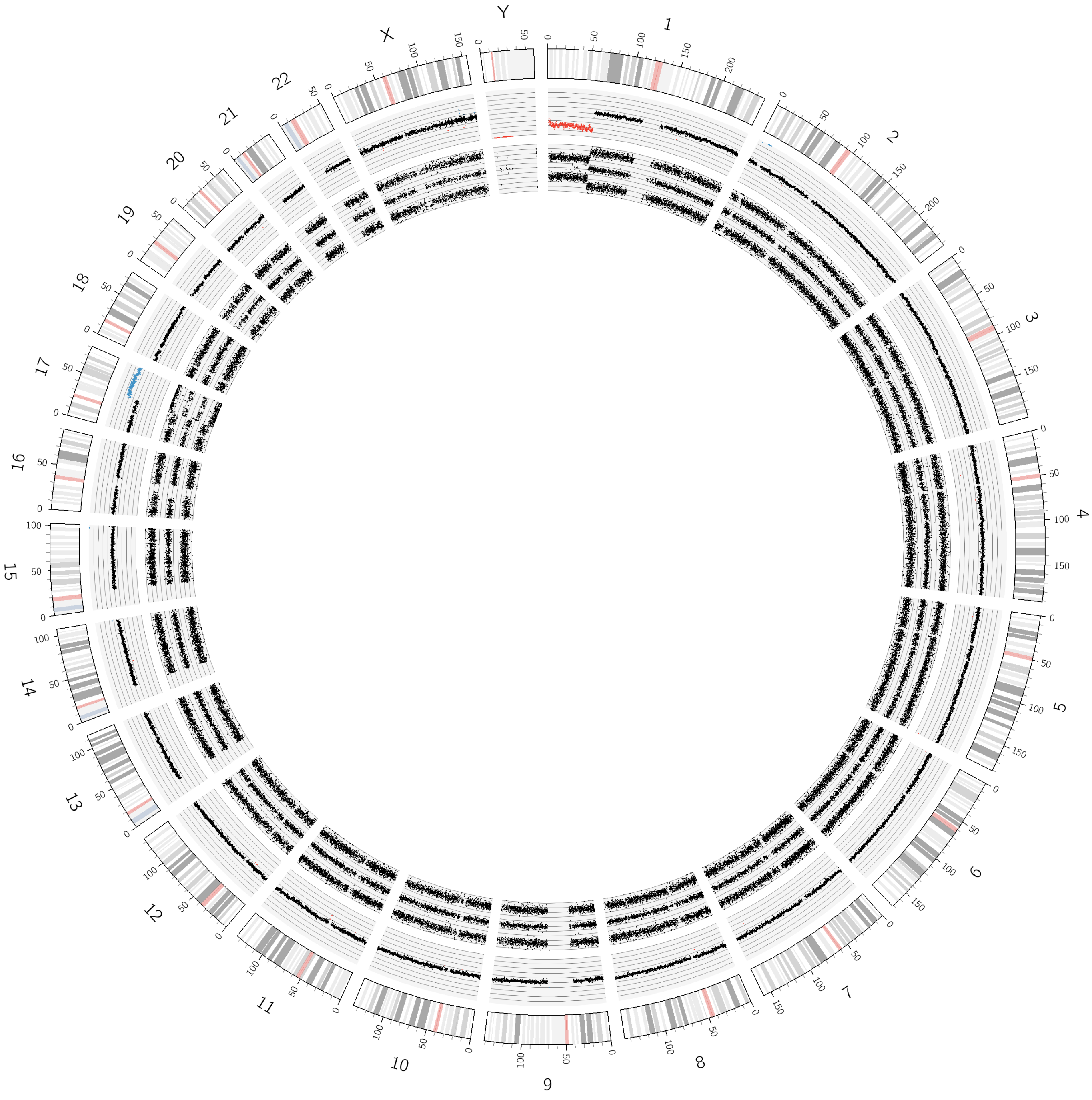

Supplementary Figure 2 displays a Circos plot of a MYCN amplified neuroblastoma. Besides the amplified MYCN locus (small blue dot on 2p), the short arm of chromosome 1 discloses a deletion (red dots) and a large portion of the long arm of chromosome 17 is gained (blue dots).
